# Childhood underweight in Ethiopia: modelling non-linear risk factors and geographic hotspots using Bayesian geoadditive methods

**DOI:** 10.3389/fpubh.2026.1760032

**Published:** 2026-04-10

**Authors:** Endeshaw Assefa Derso, Maria Gabriella Campolo, Angela Alibrandi

**Affiliations:** 1 Department of Economics, University of Messina, Messina, Italy; 2 Department of Statistics, University of Gondar, Gondar, Ethiopia

**Keywords:** BayesX, Ethiopia, MCMC, P-splines, semi-parametric Bayesian analysis, spatial variation, underweight

## Abstract

**Objectives:**

Underweight in children under 5 years of age is defined as a weight-for-age *z*-score (WAZ) of less than −2 standard deviations (−2SD) from the median of the World Health Organization (WHO) Child Growth Standards (CGS). This study examines the effect of socio-demographic covariates and geographical covariates on underweight, as well as the flexible trends of metrical covariates, to identify communities at a high risk of underweight.

**Methods:**

This study utilized cross-sectional data on underweight from the 2016 Ethiopian Demographic and Health Survey (EDHS). A Bayesian geoadditive Gaussian regression model was used to analyse a sample of 10,641 children. Appropriate prior distributions were established for the scale parameters in the models, and the inference was conducted within a fully Bayesian framework using Markov chain Monte Carlo (MCMC) simulation.

**Results:**

The results indicate that the effects of metrical covariates, such as child age, the mother’s body mass index (BMI), and maternal age, on underweight were non-linear. Specifically, the relationship between the mother’s BMI and her child’s underweight appears to be an inverted U-shape within the maternal BMI range between 12 and 50 kg/m^2^. Lower and higher maternal BMI are associated with more severe cases of underweight (as indicated by lower WAZ *z*-scores). There is also significant spatial heterogeneity, and based on inverse distance weighting (IDW) interpolation of predictive values, the western, central, and eastern parts of the country are hotspot areas for underweight children.

**Conclusion:**

Socio-demographic and community-based programmes should be comprehensively integrated into Ethiopian policy to combat childhood malnutrition.

## Introduction

Malnutrition is the biggest missed opportunity in global health; tackling it in all its forms is one of the greatest global health challenges (1, 2). In countries experiencing a nutrition crisis, such as Afghanistan, Somalia, Ethiopia, Kenya, Burkina Faso, Mali, Niger, and Yemen, malnutrition claims the lives of many young children (3). Consequently, improving the nutritional status of children requires a broad range of interventions (4).

Based on the WHO Child Growth Standards (WCGS), wasting, stunting, and underweight are the three main indicators of undernutrition (5). These three anthropometric variables are measured by *z*-scores. The *z*-score for each anthropometric variable (e.g., weight-for-age) for the *i*-th child is determined using the *z*-score (*Z_i_*) formula, which is defined as:


$$Z_{i}=\frac{{\mathrm{AI}}_{i}-{\mathrm{MI}}_{i}}{\mathrm{SD}},$$

AI refers to an individual anthropometric index (such as weight at a given age), whereas MI refers to the median of a reference population, and SD refers to the standard deviation of a reference population.

Children are stunted if their height-for-age *z*-score (HAZ) is less than negative two standard deviations (−2SD) from the median established by the WHO Child Growth Standards (WCGS) based on their *z*-scores. Similarly, underweight occurs if the weight-for-age *z*-score (WAZ) is less than −2SD from the reference median, while wasting occurs if the weight-for-height *z*-score (WHZ) is less than −2SD from the reference median (6). Although stunting and wasting are also markers of malnutrition, being underweight is used as a wider measure of overall malnutrition because it considers both chronic and acute types of malnutrition. Underweight children are at an increased risk of stunting, wasting, and other health problems associated with malnutrition (7–9).

Malnutrition is the leading cause of underweight (10). Globally, an estimated 101 million (16%) children under 5 years of age were underweight in 2011, with 26.6% of them located in Africa. Furthermore, according to the 2016 Ethiopian Demographic and Health Survey (EDHS), the prevalence of underweight was 24%. Even though there have been improvements in malnutrition trends over the past 15 years, 28% of child deaths in Ethiopia are associated with undernutrition (11). Goal 2 of the Sustainable Development Goals (SDGs) is aimed at achieving zero hunger but is falling short of its targets for ending all forms of malnutrition by 2030 (12).

The cause of being underweight is not only a lack of food. It is the result of a complex set of variables (13–18). Underweight in children has a significant association with metrical covariates such as the child’s age, mother’s age, and mother’s BMI (19–21). Most previous studies have assumed linear relationships and did not explore potential non-linear effects of the child’s age, mother’s age, and BMI. In contrast, our study applies a Bayesian geoadditive Gaussian model to the 2016 EDHS, allowing for (i) flexible non-linear modelling of child age, maternal age, and maternal BMI, and (ii) explicit estimation of spatial effects at the cluster level. This approach provides more detailed insights into how risk varies across different covariates and within the Ethiopian landscape. It identifies geographic hotspots that may inform targeted interventions. Similarly, geographic location and socio-economic characteristics were also found to be significantly associated with underweight (22–24). Lower malnutrition rates in urban areas can be attributed to a series of more favourable socio-economic conditions, which in turn lead to better care practices for children and their mothers (17).

Based on various previous studies conducted on underweight, most researchers have utilized a frequentist approach (8, 25, 26). Consequently, the frequentist approach relies solely on the data to make statistical inferences and often neglects prior knowledge about the parameters. Incorporating prior knowledge can lead to more informed predictions about the parameters than what could achieved with data alone. However, Bayesian regression allows the prior incorporation of information and permits more flexible model specifications. This flexibility can help improve the accuracy of the model and provide better insights into the underlying relationships between variables (27–29) and represents a powerful approach to disease mapping (28, 30). Furthermore, the Bayesian semi-parametric geoadditive model replaces traditional linear predictors with more flexible additive predictors, enabling the joint estimation of non-parametric metrical covariates with spatial effects (27, 31). This approach has several advantages over other statistical methods. First, it facilitates the integration of prior knowledge or beliefs into the analysis. Second, it allows for the propagation of uncertainty from the parameters to the prediction. Third, it provides a mechanism for quantifying evidence in favour of different models or hypotheses (29, 32, 33).

Although studies have examined how socio-economic factors affect underweight in classical models, little is known about the Bayesian framework incorporating spatial distribution (34–36). We developed a Bayesian approach to geoadditive regression that can estimate the effect of socio-economic covariates and spatial influences, based on identified risk factors. Our study mainly focuses on the non-linear effect of metrical covariates and spatial influences, using data from the 2016 EDHS database. We aimed to assess the spatial variation and non-linear effects of children’s age, maternal age, and maternal BMI on underweight (WAZ) among Ethiopian children under five, using a Bayesian geoadditive Gaussian model. The findings from this study may aid future research in developing more flexible models for understanding the impact of metrical variables on underweight. Additionally, the study provides guidance for policymakers seeking solutions to underweight issues.

## Methods

### Data sources

We used the data obtained from the 2016 Ethiopian Demographic and Health Survey (2016 EDHS), where geographic coordinates, sociodemographic information, and anthropometric data were collected. The 2016 EDHS used a stratified, two-stage sampling design. First, every region was split into urban and rural areas, giving a total of 21 groups (strata). Within each group, enumeration areas (EAs) were then selected in two stages. To ensure the sample reflected all lower administrative levels, the list of EAs in each group was ordered by the local administrative units before selection. At the first stage, EAs were selected using probability proportional to size, so larger areas had a higher chance of being chosen. In the first stage, 645 EAs (202 in urban and 443 in rural areas) were selected using a probability proportional to size (based on the 2007 Ethiopia Population and Housing Census) and independent selection in each stratum. In the second stage of selection, a fixed number of 28 households per cluster were selected with an equal probability systematic selection from the newly created household listing. All women aged 15–49 and all men aged 15–59 who were either permanent residents of the selected households or visitors who stayed in the household the night before the survey were eligible to be interviewed.

In each selected household, mothers aged 15–49 were interviewed, and anthropometric measurements were taken on all children under the age of 5 years in the family. We took 10,641 children in 645 clusters of 11 regions of the country, and the cluster located in a particular region was representative of that region. The detailed methodology for the 2016 EDHS survey is reported elsewhere (37).

We used a spatial framework for mapping, hotspot detection, and interpreting regional clustering in underweight. Ethiopia is a landlocked country in the Horn of Africa, extending approximately between 3° and 15° N latitude and between 33° and 48° E longitude. It borders Eritrea to the north, Djibouti and Somalia to the east, Kenya to the south, and South Sudan and Sudan to the west. Its physical geography is strongly structured by the Ethiopian Highlands, split by the Great Rift Valley, creating sharp contrasts between cooler, densely settled highlands and hotter lowlands (including the Danakil Depression). Administratively, it comprises the regional states of Tigray, Afar, Amhara, Oromia, Somali, Benishangul-Gumuz, Gambella, Harari, Sidama, Central Ethiopia, South Ethiopia, and South West Ethiopia Peoples’ Region, plus the chartered cities of Addis Ababa and Dire Dawa (36).

### Study variables

The UNICEF determinants framework for maternal and child nutrition guided the selection of explanatory variables, emphasizing immediate (diet/disease), underlying (food security, caregiving, health services, and household environment), and basic/contextual determinants (socio-economic and geographic context) (38). Furthermore, we relied on empirical evidence from Demographic and Health Survey (DHS)-based analyses and recent multi-level studies in Ethiopia that found consistent links between child growth outcomes (e.g., stunting and undernutrition) and child, maternal, household, and community factors (37, 39, 40).

Our study began with the consideration of covariates that have been identified as the key determinants of childhood malnutrition based on previous studies. The primary outcome was the continuous weight-for-age *z*-score (WAZ), calculated using the WHO Child Growth Standards. We modelled WAZ as a Gaussian response. For descriptive purposes, we also defined a binary indicator of underweight (WAZ < −2 SD), but this binary variable was not used as the response in the main geoadditive model (see Table 1).

**Table 1:** Variables in the dataset on childhood underweight in Ethiopia.

Covariate	Description
Cage_month	Child’s age in months
Region	Region where the mother lives
Mother_BMI	Mother’s BMI
Mother_age	Age of the mother in years
Mother_education	Mother’s education in categories “No education,” “Primary,” “Secondary,” and “Higher”
Child’s sex	Child’s sex, with categories “male” and “female”
Availability of electricity	Availability of electricity, with categories “yes” and “no”
Sex of household head	Sex of household head, with categories “male” and “female”
Diarrhoea_level	Child’s diarrhoea status with categories “yes” and “no”
Anaemia_level	Child’s anaemia level, with categories of “Severe,” “Moderate,” “Mild,” and “No anaemia”
Place of residence	Place of residence, with categories “urban” and “rural”

### Statistical analysis

We assigned reasonable priors (weakly informative or diffuse priors) for spatial and unknown non-linear smooth functions and fixed-effect parameters. The smooth functions capture the non-linear relationship between the continuous covariates and the response variable, modelled using a P-spline, and the spatial effect accounts for the spatial correlation and are modelled using a Markov random field (38–40). The inference was fully Bayesian and employed efficient Markov chain Monte Carlo (MCMC) simulation. For improved visualization of the non-linearity of metrical covariates on underweight, we used the Yeo-Johnson transformation, a power method used in statistics to improve the accuracy and reliability of models, especially when dealing with non-linear models (37). For model fit comparison, the deviance information criterion was employed (41).

We tried to examine the non-linearity of metrical covariates and the geographical differences of underweight in Ethiopia. The Bayesian geoadditive models are used to estimate the non-linear smooth functions of quantitative (metric) variables and to model spatial effects (42, 43). Thus, for observations ( $y_{i,}x_{i,}\omega_{i}$ ), *i* = 1, …, 10,641 continuous responses *y*, a vector of metrical covariates *x* = ${\left(x_{1}\ldots x_{p}\right)}^{\prime }$ , and a vector of fixed covariates, $\omega ={\left(\omega_{1}\ldots \omega_{p}\right)}^{\prime }.$ Given the generalized additive model (GAM) for cross-sectional data, the model includes additional predictors of the form:


$$\eta_{i}=f_{1}\left(x_{i1}\right)+\ldots +f_{p}\;\left(x_{ip}\right)+\omega_{i}^{\prime }\gamma$$ (1)

We include an additional spatial effect $f_{\mathrm{spat}}$ to the predictors of Equation 1, leading to geoadditive models (39). Therefore, by considering all non-linear effects of metrical covariates, categorical and spatial covariates for underweight collectively and replacing Equation 1 with a more flexible additive model, we get the general form of the Bayesian geoadditive model as:


$$\eta_{i}=f_{1}\left(x_{i1}\right)+\ldots +f_{p}\;\left(x_{ip}\right)+f_{\mathrm{spat}\left(\mathrm{si}\right)}+\omega_{i}^{\prime }\gamma$$ (2)

where $f_{1,}$ … $,f_{p}$ are unknown smooth pth-degree of polynomial functions of metrical covariates, and the linear combination of $\omega_{i}^{\prime }\gamma$ corresponds to the usual parametric part of the predictor, including an intercept term, and the function $f_{\mathrm{spat}}$ represents the geographical effects of spatial variable s ϵ {1,…, *S*}, indicating regions in a country. The spatial effect was specified as a proxy for numerous unobserved factors with geographic information or that were not covered by observable covariates, some of which may have a strong spatial structure and others that are only present locally. Given the covariates and unknown parameters, we assume that the response variable $y_{i}$ is a Gaussian distribution with a common variance $\sigma^{2}$ across observations for *i* = 1…10,641.

In Equation 2, the unknown function *f_j_* for various function evaluations $f_{j}\;$ = ${\left(f_{1}\;\left(x_{i1}\right)+\ldots +f_{p}\;\;\left(x_{ip}\right)\right)}^{\prime }$ can be expressed as the matrix product of the design matrix $X_{j}$ and an unknown parameter vector $\;\beta_{j}$ (Equation 3):


$$f_{j}=X_{j}\beta_{j}$$ (3)

In Bayesian geoadditive models, all the unknown parameters, $f_{1}\ldots ..f_{p},$ $f_{\mathrm{spat}},$ and an uncertain parameter γ and $\delta^{2}$ are random variables and must be assigned with an appropriate prior distribution (34, 44). Statistical analysis and graphics were done using R version v4.3.0 in the BayesX and R2BayesX packages (48, 49), and maps were produced using the open-source package QGIS v1.8.0.

## Results

In this study, categorical variables with fixed effects, non-linear metrical covariates, and spatial covariates were investigated. The Bayesian framework treated all of them and assigned suitable priors with varied forms and levels of smoothness. To summarize the covariate features, the mean weight-for-age *z*-score of 1.28 standard deviations. In our settings, 81.45% of the children and mothers reside in rural regions, while the remaining 18.55% dwell in urban areas. Approximately 6,838 (64.27%) of mothers have no education; 2,678 have completed their primary education; 734 have completed secondary school; and 391 have completed their secondary education. Furthermore, of all the children considered in the 2016 EDHS data, 8,826 had diarrhoea in the 2 weeks preceding the survey, whereas only 1815 did not have diarrhoea during the survey.

**Figure 1:**
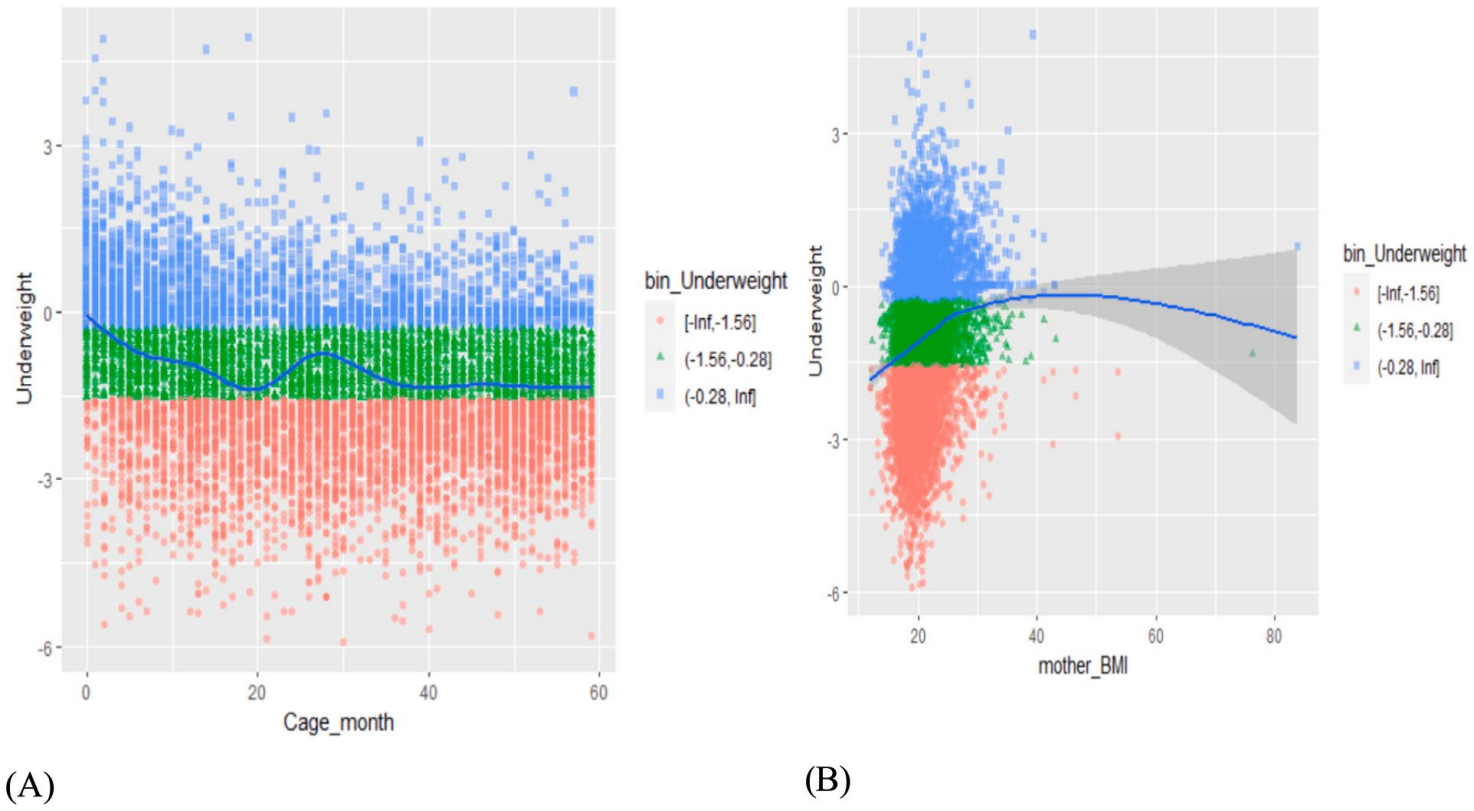
Yeo-Johnson transformation visualization of **(A)** child’s age vs. underweight; **(B)** mother’s BMI vs. underweight.

Apart from the descriptive statistics, the Yeo–Johnson transformation (Figure 1) visualizes a range of values that were used to group data into categories for the purpose of detailed visualization (37). Based on the binned visualization of child age and underweight, underweight does not steadily increase or decrease with age (see the left part of Figure 1). The plot oscillates back and forth from the child’s age between 20 and 40 months and then decreases steadily on the right. Similarly, being underweight in children does not follow an increasing or decreasing trend for each value of the mother’s BMI (see the right part of Figure 1). Therefore, from the Yeo-Johnson transformation visualization, the metrical covariates such as the mother’s BMI, the child’s age, and the mother’s age at birth are good candidates for the non-linear effects on children’s underweight status based on the EDHS 2016 dataset.

**Figure 2:**
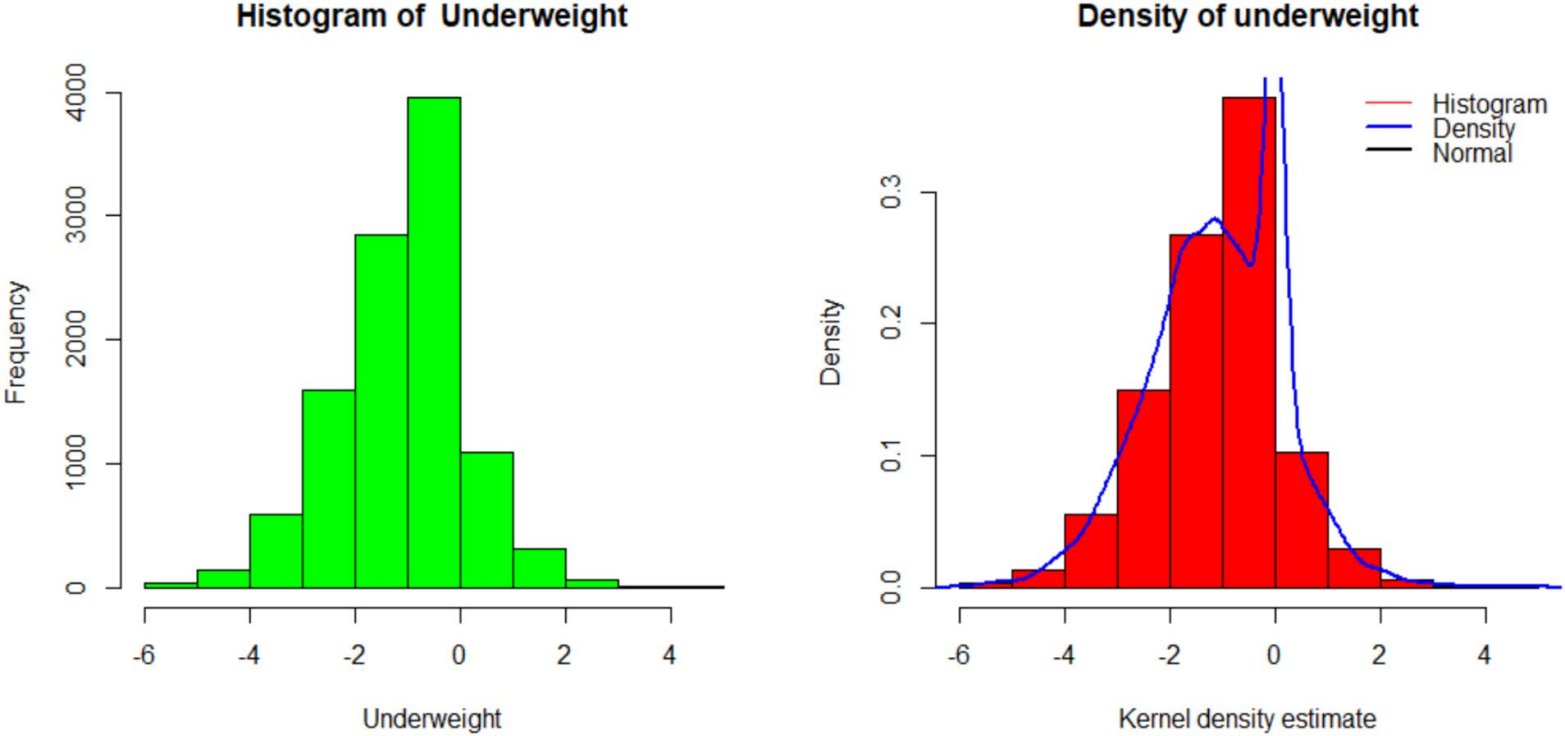
Histogram (left) and kernel density estimates (right) of the underweight.

A histogram and kernel density estimates of the *z*-score distribution, as well as a normal density curve with its corresponding mean and variance estimated from the sample of underweight children, are shown in Figure 2. This demonstrates that a Gaussian model is appropriate for our inference.

We employed the Bayesian geoadditive model with the BayesX stepwise selection method to identify various covariates that influence underweight. The fixed effects and smooth term variances of the geoadditive Gaussian model are presented in Tables 2, 3. From the tables, the 50, 2.5, and 97.5% quantiles represent the median, lower, and upper bounds of a 95% credible interval, respectively. They quantify uncertainty that can be interpreted as a range within which, with 95% posterior probability, the true value of the parameter lies, and if a parameter’s credible interval excludes zero, the parameter is considered statistically significant (45, 46).

**Table 2:** The posterior estimates of the fixed effects parameter for underweight in Ethiopia based on the 2016 EDHS.

Estimates of fixed effects, parametric coefficients					
Variables	Mean	SD	2.5% quantiles	50% quantiles	97.5% quantiles
Intercept	−1.1037	0.3060	−1.7565	−1.0935	−0.5333
Sex of child					
Male (Ref.)					
Female^a^	0.0837	0.0225	0.0371	0.0840	0.1293
Residence					
Urban (Ref.)					
Rural^a^	−0.0296	0.0451	−0.1160	−0.0302	−0.0110
Availability of electricity					
Yes (Ref.)					
No^a^	−0.1976	0.0418	−0.2746	−0.1977	−0.1158
Sex of household head					
Male (Ref.)					
Female^a^	−0.0521	0.0294	−0.1098	−0.0525	0.0074
Diarrhoea level					
No					
Yes^a^	0.2161	0.0317	0.1534	0.2174	0.2780
Anaemia level					
Not anaemic (Ref.)					
Severe^a^	−0.5319	0.0533	−0.6323	−0.5343	−0.4189
Moderate^a^	0.2998	0.0239	0.2529	0.2988	0.3456
Mild	0.0139	0.0276	−0.0421	0.0146	0.0663
Mother’s education					
No (Ref.)					
Primary^a^	−0.0946	0.0265	−0.2796	−0.0941	−0.0424
Secondary^a^	0.1232	0.0375	0.0441	0.1241	0.1939
Higher^a^	0.2277	0.0265	0.2796	0.2272	0.1754

Ref.: reference group.

a Indicates that the effects are statistically significant at the 95% confidence level.

**Table 3:** Posterior estimates of the smooth term variances and scale estimate for underweight in Ethiopia (summary statistics of the MCMC samples for the smoothing parameters) based on 2016 EDHS data.

Smooth term variances							
Variables	Mean	SD	2.5%	50%	97.5%	Min	Max
sx (child age/month)*	1.6961	1.2180	0.4691	1.3796	4.8556	0.3018	14.9313
sx (spatial effect)*	0.3047	0.1819	0.0721	0.2690	0.7589	0.0392	1.7381
sx (mother’s age)*	0.0114	0.0181	0.0007	0.0055	0.0604	0.0003	0.1588
sx (mother’s BMI)*	0.4226	0.9056	0.0322	0.1890	2.1381	0.0142	11.4281
Scale estimate							
Sigma2	1.3511	0.0187	1.3154	1.3510	1.3892		

*N* = 10,641, burn-in = 2000, method = MCMC, family = Gaussian, iterations = 12,000, step = 10, ^*^significance.

The results of the fixed effects are as expected. Female children, children from households without electricity, and rural residents had diarrhoea, severe and moderate anaemia, and maternal and primary, secondary, and higher education were statistically significant at the 5% level. However, being a female head of household was not statistically significant (Table 2). Therefore, the findings suggest that female children are at greater risk of being underweight than male children. Children born to mothers with secondary and higher educational levels and who had access to electricity in their household were at the lowest risk of underweight compared to children born to mothers with primary and lower educational levels and who could not access electricity.

Furthermore, the posterior mean of the effect of female children on underweight is 0.0837 with a standard deviation of 0.023 compared to the reference group (male children). These results suggest that the effect of female children on underweight is likely positive and relatively precise and is approximately 0.0837 units higher than that of male children on average. Additionally, the 2.5, 50, and 97.5% quantiles of the posterior distribution of the effect of female children are 0.0371, 0.0840, and 0.1293, respectively. Similarly, the effect of the absence of electricity on underweight is likely negative and is approximately −0.1976 units from the reference level (the presence of electricity) on average, with a 95% probability that the true effect lies between −0.2746 and −0.1158 units.

Table 3 represents the posterior variance parameters associated with the smooth terms. It shows the posterior mean of the variance, the estimated standard deviation of the variance, the 2.5 and 97.5% quantiles of the posterior variance distribution, and the median of the posterior distribution of the variance for spatial and metrical covariates. The metrical covariates children’s age, mother’s BMI, and mother’s age show significant predictors of underweight in Ethiopia. It also reveals that the spatial variance component is significant, implying that socio-economic factors are unable to explain a substantial portion of this regional heterogeneity.

Considering the metrical covariates, the estimated mean of the variance, the posterior standard deviation of the variance, and the 50% quantile of the posterior median of children’s age are 1.6961, 1.2180, and 1.3796, respectively. Besides, the 2.5 and 97.5% quantiles of the posterior variance distribution for children’s age are 0.05 and 0.15, respectively. The posterior mean of the spatial random effect variance [sx(spatial effect)] is 0.3047. This indicates that there is spatial variation in underweight that is not explained by the other covariates in the model. This table also shows the hyperparameter of error variance (Sigma2), which is the amount of variation in the response variable that is not explained by the covariates and the spatial effects. A smaller value of error variance indicates that the model is able to explain a large portion of the variance in the response variable (47). In conclusion, this output indicates that the estimated posterior mean of Sigma2 is 1.3511, with a relatively small standard deviation of 0.0187 and a 95% credible interval; the scale parameter Sigma2 is well identified.

**Figure 3:**
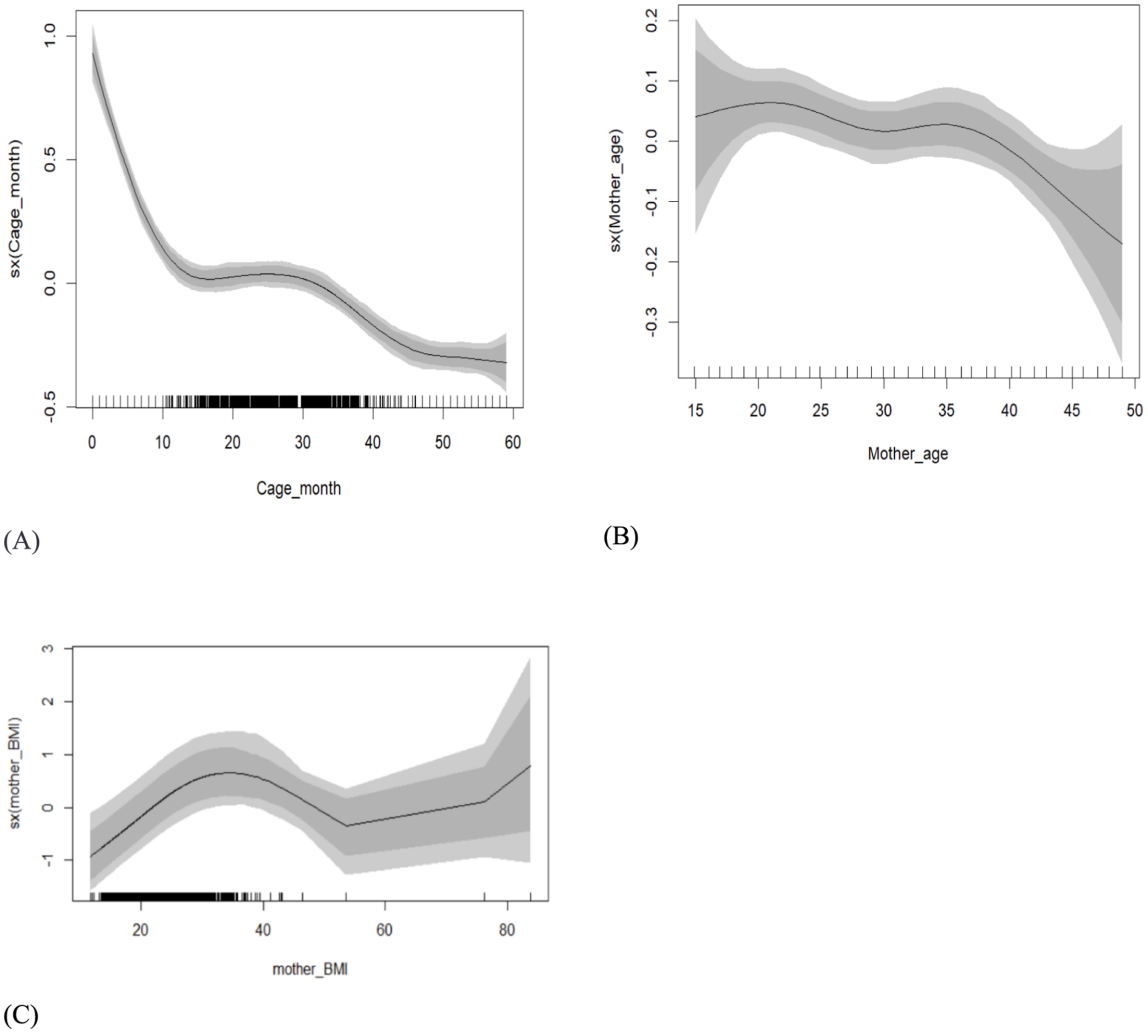
The estimated non-linear effects of metrical variables on underweight in Ethiopia, together with the posterior mean and the 80 and 95% credible intervals. The panels **(A–C)** represent the effects of the child’s age, mother’s age, and mother’s BMI on underweight, respectively.

Figure 3 depicts the posterior means as well as the 95 and 80% credible intervals of metrical variables. The 95% credible interval indicates the range of values that the true variance is likely to fall within with 95% probability, while the 80% credible interval provides a narrower range of values with 80% probability, and the wider the shaded area, the more uncertain the predictions are (46).

The top-left panel of Figure 3 shows that child’s age has a non-linear impact on underweight. Particularly between the ages of 0 and 10 months, the child’s underweight gradually worsens, following an almost linear pattern. However, after 10 months, the tendency changes and stabilizes at a moderate level at approximately 30 months. As shown in the graph, the relation between the mother’s BMI and her child’s weight-for-height *z*-score appears to be an inverted U-shaped curve with maternal BMI below 50. However, higher maternal BMI seems to have a significant impact on the children’s underweight. Furthermore, a maternal BMI of less than 18.5 is considered underweight, and it indicates acute undernutrition in the mother. This may lead to negative health outcomes for both the mother and child, such as an increased risk of complications during pregnancy and childbirth and a higher risk of developmental delays for the child who is underweight. Finally, the effects of mother age on underweight are relatively small (see top right panels of Figure 3). It shows that the weight-for-age *z*-score is high for mothers between 15 and 35 years. Then, after the age of 35 years, the *z*-score of weight-for-age decreases (underweight increases), and the effects of mothers’ age increase with an almost linear trend on underweight. Furthermore, the plotted line corresponds to the average predicted response across the predictor values, and tick marks on the *x*-axis correspond to the unique predictor values in the selected dataset.

**Figure 4:**
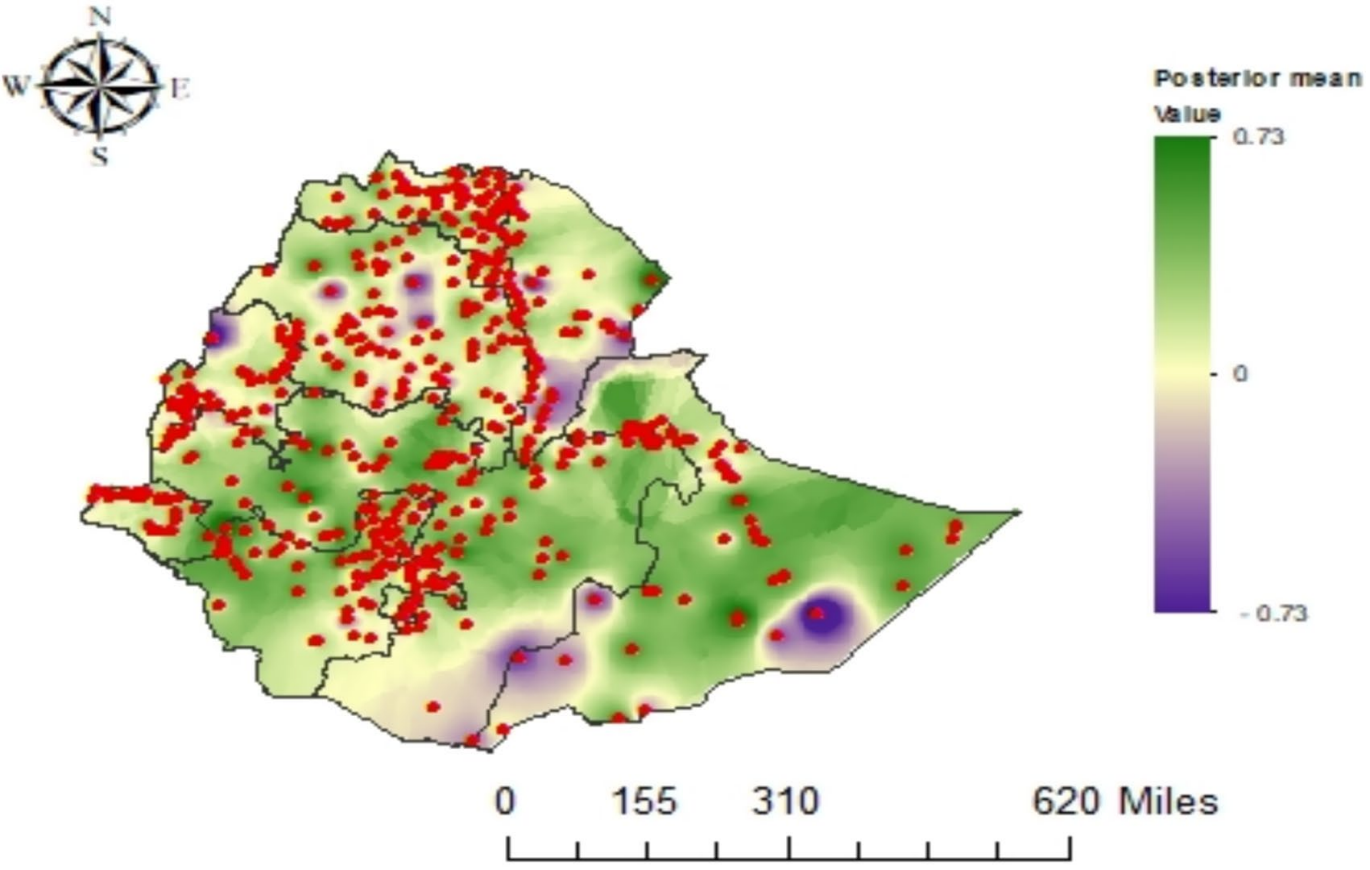
The Gaussian model’s posterior mean of the spatial effects of underweight.

The posterior spatial effect of the fitted model in Ethiopia is depicted in Figure 4. The posterior probability maps demonstrate the relevance of the spatial effects (the red/blue colour in the map indicates the significance level and whether it has a positive or negative influence on the *z*-score, and grey indicates non-significance). This illustrates that there is solid evidence for spatial variation of child underweight under Gaussian modelling, and most sites in Ethiopia revealed significant spatial effects on underweight. Furthermore, the spatial effects are centred on zero; the average *z*-score across all areas is zero, but the overall level is determined using the intercept term.

**Figure 5:**
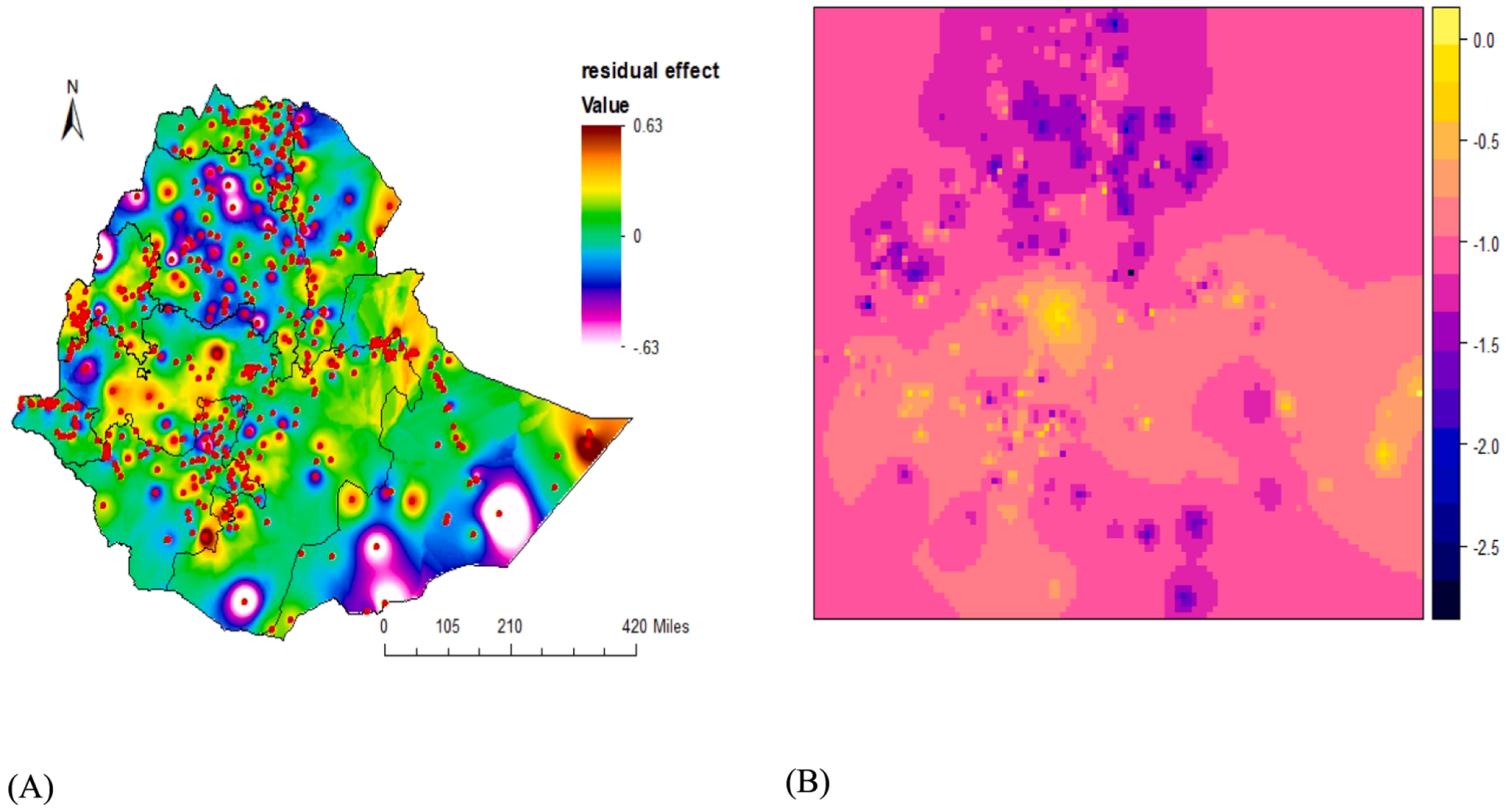
Posterior mean of the residual spatial effects for underweight under the Gaussian model **(A)** and IDW-interpolated predicted values **(B)**.

The residual spatial pattern from the Gaussian model (left panel of Figure 5) and the IDW interpolation of predicted values (right panel of Figure 5) are shown in Figure 5. This observed residual spatial pattern in underweight children may be attributed to unobserved variables not reflected by the covariates in the models, and their identification remains a matter of hypothesis. Furthermore, the IDW interpolation of predicted values provides estimates of underweight to identify high-risk areas at unsampled locations, to evaluate the effectiveness of intervention strategies, and to facilitate interpretation (48). Therefore, the yellow colour in the IDW interpolation of predicted values of the figure indicates a higher level of underweight and represents a hotspot area (the left plot). The prediction for unsampled locations was performed using the observed values of the nearest locations.

**Figure 6:**
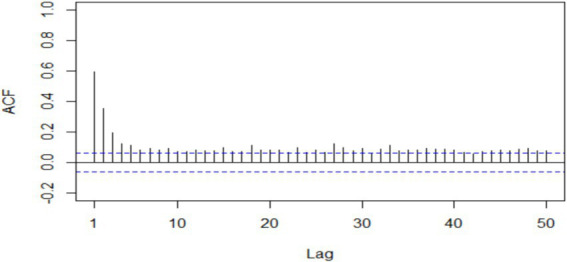
Maximum autocorrelation of all model parameters for underweight in under-5 Ethiopian children.

The goodness of fit of a Bayesian geoadditive model can provide insights into the accuracy and precision of the model’s predictions. Trace plots, autocorrelation plots, and residual plots are important diagnostic tools for Bayesian geoadditive modelling (49). Autocorrelation is a useful tool for assessing the goodness of fit of a Bayesian geoadditive model and for gaining a comprehensive understanding of the model’s performance (39, 50, 51). It is a measure of the correlation between a parameter value at iteration *t* and the corresponding value at iteration *t + k* (52). The plot depicted in Figure 6 shows a rapid decay in autocorrelation as iteration *k* increases. Slight autocorrelation is visible between the current and previous iterations. However, this is not a major concern, as it does not appear to have a significant impact on the mixing or convergence of the chains. Moreover, the autocorrelation drops off quickly, indicating that the MCMC algorithm is efficiently exploring the posterior distribution and is mixing well. This low autocorrelation is an indication of shorter convergence times and unbiased inference (39, 50).

**Figure 7:**
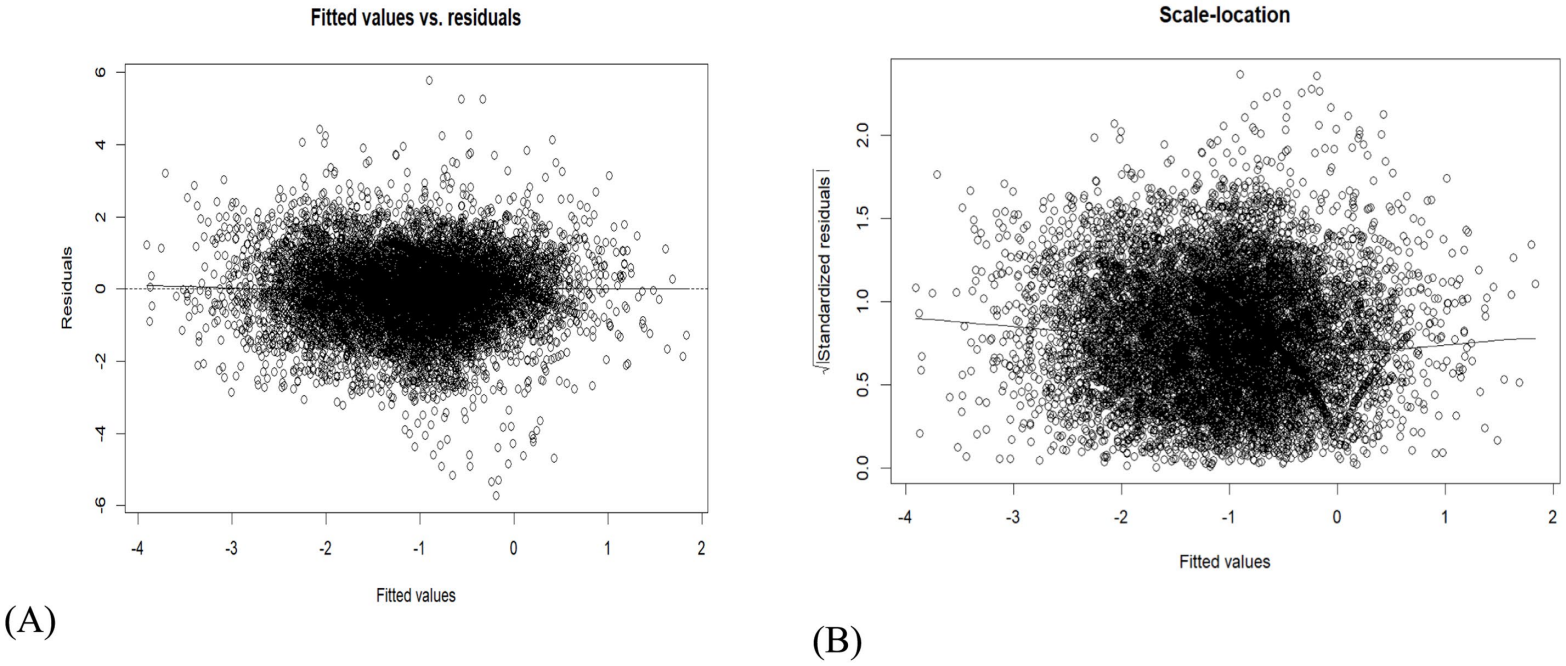
The residual plot **(A)** and the scale-location plot **(B)**.

Scale-location and residual plots are also diagnostic tools that can help identify potential issues with a model’s fit and convergence (39, 53, 54). The scale-location plot (the right panel of Figure 7) is randomly scattered around zero; therefore, the model is appropriate for the data. Similarly, the residual plot depicted on the left assesses the adequacy of the model fit and identifies any patterns in the residuals that may suggest model misspecification. The fitted values are the model’s predictions for the response variable across different spatial units, whereas the residuals are the discrepancies between the observed and predicted values. When the model is correctly specified, a random dispersion of points is expected (34, 55). Thus, the residuals (see the right panel of Figure 7) exhibit no visible patterns in the data that would suggest a poor fit.

## Discussion

In this nationally representative sample of Ethiopian children under five, we found that children in urban areas are less likely to be underweight than their rural counterparts, and this finding is robust. A better quality of healthy environment and sanitation is present in urban regions. However, living in rural areas is associated with several disadvantages, such as poor health, a lack of access to clean water, a deficiency in milk intake, and poor personal cleanliness. According to the findings of the study, the place of living has a major influence on being underweight. This result contradicts the findings of (56), who reported that the residence has no significance for the child’s underweight; however, (17, 58) studies conducted in Tanzania and Malawi reported a significant association between urban residence and child underweight. Likewise, female children had a lower likelihood of being underweight than male children. This result confirms findings from previous studies (57, 58). Gibson, however, reported no significant gender differences in underweight in Papua New Guinea (59).

Maternal education is a basic determinant of childcare knowledge and behaviours. In our study, mothers’ educational attainment had a substantial influence on child underweight and reduced the risk of malnutrition. This study supports the idea that a mother who has received an education is more responsible for delivering a sick child to medical treatment. In addition, the amount of time mothers spend discussing their child’s sickness with a doctor is proportional to their level of education. Uneducated women with sick children benefit far less from medical consultations than educated women. Our findings suggest that maternal education has a significant impact on children’s underweight, which is consistent with studies conducted in underdeveloped countries (60, 61).

The BMI of a woman influences her capacity to effectively carry, birth, and care for her children. Malnutrition occurs when a non-pregnant woman’s BMI falls below the recommended cutoff point (approximately 18.5 kg/m^2^). Women who are malnourished may deliver an underweight child, implying that there is a link between the mother’s BMI and child nutritional status. According to our results, the relationship between the BMI of mothers and the children’s weight-for age *z*-score appears to follow an inverted U-shape for BMI values between 12 and 50. Both high and low maternal BMIs seem to have a significant association with increased underweight. This result contradicts (62), which reported that all metrics exhibited roughly linear trends with positive slopes.

The effects of maternal age on underweight are quite clear (see the top right panels of Figure 2). It shows that the weight-for-age *z*-score is high for mothers aged between 15 and 35 years. After the age of 35, the *z*-score of weight-for-age decreases (underweight increases). This indicates that their children are better off in terms of nutritional status compared with children whose mothers are in the younger age group. This is because, as women age, they are more likely to have chronic health conditions, such as diabetes, hypertension, or heart disease, which can affect the health of the developing foetus and increase the risk of underweight (63, 64), and older mothers may be more likely to have unhealthy lifestyle habits, such as smoking, drinking alcohol, or poor nutrition, which can increase the risk of underweight in babies (65, 66). Therefore, our results contradict the study conducted by (62), which reported that mothers under the age of 20 have a greater effect on their children being underweight.

Child’s age also has a non-linear trend in underweight. Particularly, the child’s underweight gradually worsens in an almost linear pattern at ages below 10 months. However, after 10 months, the tendency changes and then stabilizes at a moderate level up to approximately 30 months. This means that, while the risk of malnutrition remains, it does not grow as fast as it did previously. It may be that younger children are more sensitive to underweight owing to a lack of access to proper food and healthcare, while older children are more likely to be influenced by social and environmental variables such as poverty and food insecurity (67). Hence, the results are consistent with other researchers’ findings that child age affects underweight non-linearly (31, 58, 62). The study results also show that children living in western, central, and eastern Ethiopia, as well as some other regions in northern Ethiopia, have underweight problems.

## Conclusion

This study addresses underweight among children under 5 years using a Bayesian geoadditive model. According to this analysis, factors such as the mother’s education, the mother’s and child’s residence, the child’s diarrhoea and anaemia status, child sex, and the availability of electricity were found to be significant. However, the effects of the sex of the household head are negligible. Our analysis also supports the flexible modelling of metrical factors (child’s age, mother’s BMI, and mother’s age), and attention should also be given to unmeasured factors on childhood underweight at the community level, especially in central and eastern Ethiopia, which exhibited hotspot spatial effects. Socio-demographic and community-based programme development should be considered comprehensively in Ethiopian policy to combat childhood malnutrition.

## Data Availability

Publicly available datasets were analysed in this study. This data can be found at: the data is available on correspondence author based on request.
